# Meaning and Use in the Expression of Estimative Probability

**DOI:** 10.1162/opmi_a_00066

**Published:** 2022-11-30

**Authors:** Bob van Tiel, Uli Sauerland, Michael Franke

**Affiliations:** Faculty of Philosophy, Theology and Religious Studies, Radboud University Nijmegen, The Netherlands; Leibniz-Zentrum Allgemeine Sprachwissenschaft, Berlin, Germany; Department of Linguistics, University of Tübingen, Germany

**Keywords:** probability, language, pragmatics, semantics, computational model

## Abstract

Words of estimative probability (WEPs), such as ‘possible’ and ‘a good chance’, provide an efficient means for expressing probability under uncertainty. Current semantic theories assume that WEPs denote crisp thresholds on the probability scale, but experimental data indicate that their use is characterised by gradience and focality. Here, we implement and compare computational models of the use of WEPs to explain novel production data. We find that, among models incorporating cognitive limitations and assumptions about goal-directed speech, a model that implements a threshold-based semantics explains the data equally well as a model that semantically encodes patterns of gradience and focality. We further validate the model by distinguishing between participants with more or fewer autistic traits, as measured with the Autism Spectrum Quotient test. These traits include communicative difficulties. We show that these difficulties are reflected in the rationality parameter of the model, which modulates the probability that the speaker selects the pragmatically optimal message.

## INTRODUCTION

Our ability to express probability is of great importance in daily and scientific life. Sometimes, we can use precise numbers when referring to probabilities; for example, we might say that the probability of a fair coin landing on heads is 50%. But very often, we do not—or cannot—know the exact probability of a particular event. In those cases, we might prefer to use what Kent (1964) called words of estimative probability (WEPs) to provide a vague estimate of the actual probability (Erev & Cohen, 1990; Juanchich & Sirota, 2019). The class of WEPs is highly diverse, ranging from simple words (e.g., ‘possible’, ‘likely’) to complex phrases (e.g., ‘more often than not’, ‘a small but real possibility’).

Because of their central importance, the meaning and use of WEPs has been studied extensively across many disciplinary boundaries (e.g., Beyth-Marom, 1982; Friedman & Zeckhauser, 2015; Kratzer, 1991; Shinagare et al., 2019; Wallsten et al., 1986). From this line of research, two radically different views on the meanings of WEPs have emerged.^1^

The first view holds that sentences containing WEPs have crisp *truth conditions* (e.g., Kratzer, 1991). According to this view, sentences with WEPs carve up the space of possibilities into those where the sentence is true and those where it is false. More specifically, many current proposals argue that WEPs denote *thresholds* on the probability scale (e.g., Lassiter, 2019; Moss, 2015; Swanson, 2006; Yalcin, 2007). Thus, the meanings of ‘a good chance’, ‘possible’, and ‘unlikely’ can be defined as follows, where ‘P(*x*)’ stands for the probability of an event *x*:

(1)  a. 〚there is a good chance that *x*〛 = [P(*x*) > P(not-*x*)]
    b. 〚it is possible that *x*〛 = [P(*x*) > 0]
    c. 〚it is unlikely that *x*〛 = [P(*x*) < P(not-*x*)]

An alternative view holds that the meanings of WEPs are gradient and centered around small areas of prototypical use (e.g., Bocklisch et al., 2012; Jaffe-Katz et al., 1989; Zimmer, 1983). This *prototype-based* approach is often couched within the framework of *fuzzy logic*. Whereas the truth-conditional view assumes that sentences with WEPs are always either true or false, fuzzy logic argues that they can be true or false to varying degrees (e.g., Zadeh, 1983, 1996).

**Figure 1.**
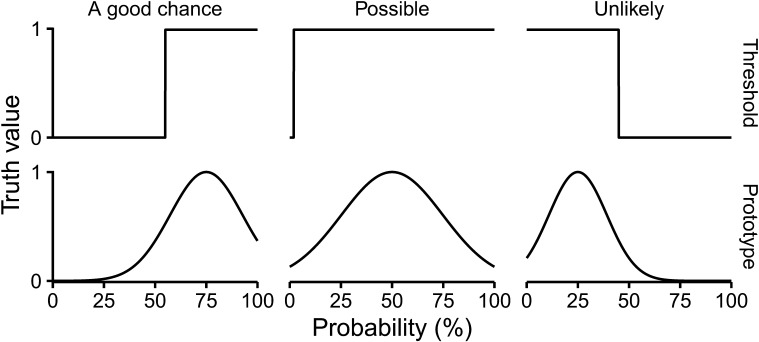
Example threshold-based and prototype-based meanings for three WEPs.

To illustrate the contrast between the two views, Figure 1 visualises hypothetical threshold-based and prototype-based meanings for three WEPs.

Apparent support for the prototype-based approach comes from experimental data on the *use* of WEPs. Invariably, such data show that people associate WEPs with gradient and focalised ranges on the probability scale (e.g., Lichtenstein & Newman, 1967; Reagan et al., 1989; Willems et al., 2020). For example, Mosteller and Youtz (1990) report that their participants associated ‘possible’ with a median probability of 38.5% and an interquartile range of 42.7%. Mosteller and Youtz interpret this finding as indicating that ‘It is possible that *x*’ implies that the probability of x lies between 17% and 60%, but most likely around 40%.

According to the prototype-based approach, such patterns of gradience and focality must be reflected in the underlying semantics of WEPs. Indeed, the prototype-based approach essentially proposes that meaning *is* use (Budescu & Wallsten, 1995; Clark, 1990). By contrast, the truth-conditional approach is *modular* in that it takes meaning to be an independent level of representation that requires a separate pragmatic module to connect to actual language use (Partee, 1999, 2001). An important challenge for the truth-conditional approach is to flesh out this pragmatic module to demonstrate how its sparse meanings give rise to complex patterns in language use.

In this paper, we show how a threshold-based semantics for WEPs gives rise to patterns of gradience and focality in their use, once the semantics is embedded within a pragmatic framework that models speaker behaviour as boundedly *rational* (cf. Frank & Goodman, 2012; Franke, 2009; Goodman & Frank, 2016). Here, ‘rational’ means that speakers prefer to produce those WEPs that are the most likely to receive the intended interpretation on the part of the hearer (cf. Grice, 1975). We show that a threshold-based approach that is embedded in such a model of pragmatic communication offers an equally compelling account of novel data on the production of WEPs as a prototype-based approach that directly encodes gradience and focality into the meanings of WEPs.

Our study builds upon an earlier study by van Tiel et al. (2021). In that study, it was shown that patterns of gradience and focality in the use of quantity words (e.g., ‘some’, ‘all’) could be reconciled with a truth-conditional view on their underlying semantics. Here, we examine whether that conclusion generalises from the quantity domain to the domain of probability.

A secondary goal of this study is to validate probabilistic pragmatic models by comparing the production behaviour of people with a low and high *autism spectrum quotient* (AQ) (Baron-Cohen et al., 2001). AQ is a quantitative measure of the extent to which individuals exhibit traits that are associated with *Autism Spectrum Disorder* (ASD). These traits include difficulties with pragmatic communication (American Psychiatric Association, 2013), though the source and scope of these difficulties have been a matter of intense debate (e.g., Baron-Cohen, 1995; Chevallier et al., 2012; Kissine, 2012, 2021). We investigate whether such self-reported pragmatic difficulties are reflected in the model parameters; specifically, in a rationality parameter that modulates the probability with which the speaker selects the pragmatically optimal message.

The next section describes our production experiment. Afterwards, we describe the computational model which we use to answer our two research questions, viz. (i) whether patterns of gradience and focality in the use of WEPs can be explained on the basis of a threshold-based semantics, and (ii) whether participants with more autistic traits are less likely to select the pragmatically optimal message than participants with fewer autistic traits.

## PRODUCTION

With some exceptions (e.g., Herbstritt & Franke, 2019; Karelitz & Budescu, 2004; Schuster & Degen, 2020), previous experimental studies have investigated how hearers *interpret* WEPs (e.g., Alstott & Jasbi, 2020; Elsaesser & Henrion, 1990; Renooij & Witteman, 1999); by contrast, in this study, we investigate how speakers naturally *produce* WEPs.

An important advantage of measuring production behaviour is that experiments that measure the interpretation of WEPs typically require participants to engage in *metalinguistic reasoning*. For example, Mosteller and Youtz (1990) asked participants to associate WEPs with ranges on the probability scale. This task requires participants to actively reflect on the meanings of WEPs, and might cause them to draw potentially artificial semantic distinctions between the WEPs that are presented throughout the experiment. As a consequence, it is unclear whether the experimental task measures meaning, use, or participants’ beliefs about meaning or use, which problematises the interpretation of the data. Here, we ask participants to describe displays, which is intuitively less likely to invite active reasoning about the meaning and use of WEPs.

**Figure 2.**
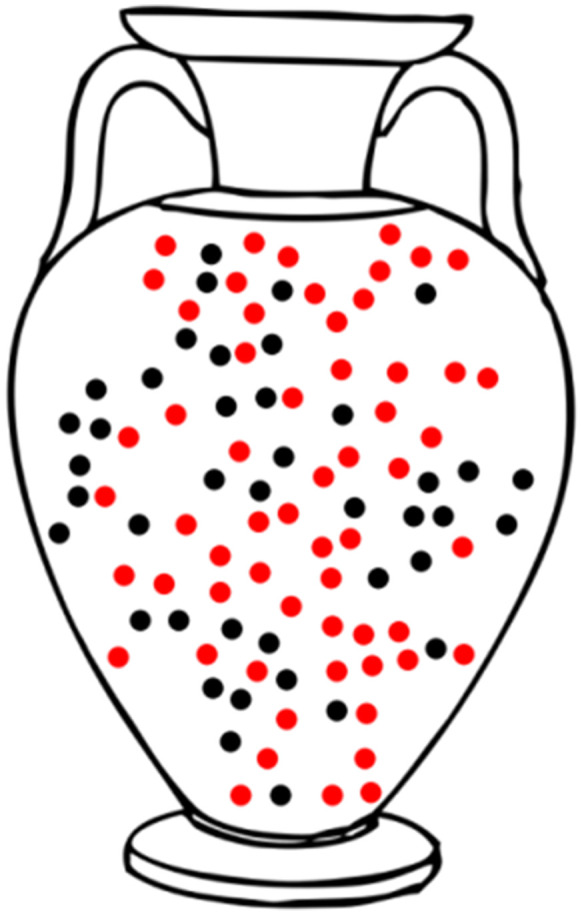
Example display used in the production experiment.

For our production experiment (Exp. 1), we recruited 255 participants on Mechanical Turk.^2^ Participants were presented with displays showing vases containing 100 randomly distributed marbles (e.g., Figure 2). The marbles were either red or black. One display was created for each of the 101 possible distributions of black and red marbles, and each participant saw a random selection of 25 displays. Participants were asked to describe these displays by freely completing the sentence frame ‘If you randomly take a marble from this vase, ______ that it is red’. Participants were instructed not to use numbers or percentages.

We used a relatively open-ended sentence frame rather than one that steered participants towards using WEPs from a specific part of speech. Our motivation for this decision was that we wanted to see which WEPs naturally come to mind, and to accommodate different response preferences observed in previous studies (Budescu et al., 1988; Karelitz & Budescu, 2004).

In total, participants produced 1,379 unique responses. Here, we analyse only the 24 WEPs that were mentioned at least 50 times, plus the prominent boundary WEPs ‘impossible’ and ‘certain’. This selection consists of 15 adjectival and 11 nominal WEPs. Similarly to previous studies, we observed distinct response patterns: out of the 185 participants who produced at least five WEPs that were included in the analysis, 50 produced exclusively adjectival WEPs; 26 only nominal ones. The remaining 109 participants produced both adjectival and nominal WEPs, suggesting that most participants naturally use a mix of both types of expressions.

**Figure 3.**
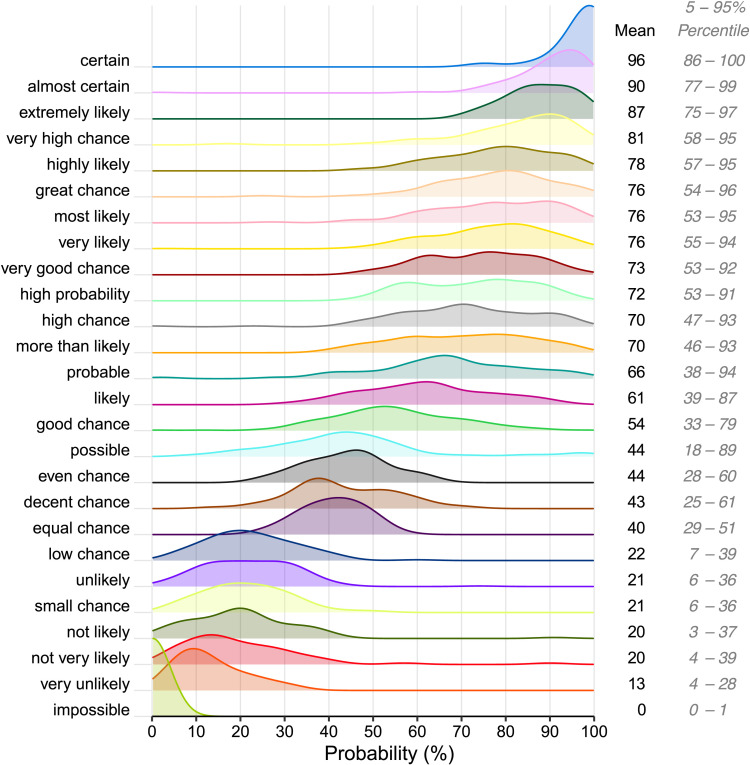
**Density plot showing the production probabilities of the most frequently produced WEPs in the production experiment.** Next to the plot are the mean probabilities in which WEPs were produced and the corresponding 5–95 percentiles.

Figure 3 shows the production probabilities of the WEPs in our sample. The results clearly show that participants associate WEPs with gradient and focalised ranges on the probability scale. Can these patterns of use be reconciled with the truth-conditional idea that sentences with WEPs are always either true or false? Or do they necessitate the incorporation of gradience and focality into the semantics of WEPs, as argued for by the prototype-based approach?

To answer these questions, we make use of the computational model of language use introduced by van Tiel et al. (2021), which in turn is based upon the more general Rational Speech Act framework (e.g., Frank & Goodman, 2012; Goodman & Frank, 2016). Here, we give a brief overview of the model.

## MODEL

The model takes as its point of departure a lexicon that associates each pair of a message *m* ∈ *M* and a state of affairs *t* ∈ *T* with a truth value. The set of messages consists of the 26 WEPs in our sample, i.e., *M* = {*m*_almost certain_, *m*_certain_, …}. The set of states consists of the 101 possible probabilities of drawing a red marble. In our design, these probabilities corresponded to the number of red marbles, i.e., *T* = {*t*_0_, *t*_1_, …, *t*_100_}.

We define and compare two types of lexica: a threshold-based lexicon that associates each WEP with a threshold on the probability scale, and a prototype-based lexicon that associates each WEP with a gradient and focalised range.

### Threshold-based Lexicon

The threshold-based approach argues that WEPs denote thresholds on the probability scale. The type of threshold associated with a WEP depends on its *monotonicity*, i.e., its inferential potential. Monotone increasing WEPs like ‘possible’ license inferences from sets to supersets, e.g., from ordering salmon to ordering fish, as shown by the validity of the argument in (2). By contrast, monotone decreasing WEPs like ‘impossible’ license inferences from sets to subsets, as shown in (3). Non-monotone WEPs like ‘possible but not certain’ license neither type of inference.

(2)  It is possible that he ordered salmon.
    → It is possible that he ordered fish.
(3)  It is impossible that he ordered fish.
    → It is impossible that he ordered salmon.

Monotone increasing WEPs place a lower bound on the probability scale; monotone decreasing ones an upper bound. Non-monotone WEPs often place both a lower and upper bound on the probability scale.

We determined the monotonicity of the WEPs in our sample by consulting our intuitions about the validity of arguments such as (2) and (3). Consequently, the following WEPs were classified as monotone decreasing: ‘not likely’, ‘not very likely’, ‘unlikely’, ‘very unlikely’, and ‘impossible’. All other WEPs were classified as monotone increasing.

Based on the foregoing, we may define a threshold-based lexicon 𝔏_TH_. This lexicon associates each message *m* with a threshold *θ*, so that the truth value of *m* in state *t* is:

$${𝔏}_{TH}\left(m,t\right)=\left\{\begin{array}{l} 1\;\text{if}\;t>\theta_{m}\;\text{and}\;m\;\text{is}\;\text{monotone}\;\text{increasing};\\ 1\;\text{if}\;t<\theta_{m}\;\text{and}\;m\;\text{is}\;\text{monotone}\;\text{decreasing};\\ 0\;\text{otherwise}. \end{array}\right.$$

The thresholds are treated as free parameters in the model, which are to be inferred from the data. We use Bayesian inference to encode prior expectations about the likely meanings of WEPs as weakly informative prior distributions over thresholds (Gelman et al., 2014).

### Prototype-based Lexicon

The prototype-based approach holds that WEPs denote gradient and focalised ranges on the probability scale. To implement this approach, we make use of *fuzzy logic*, which argues that the truth value of a sentence can take any value in the [0, 1] interval (e.g., Zadeh, 1983, 1996). Specifically, we assume that each WEP is associated with two parameters: a *prototype* and a *distance measure*.

The prototype is the state of affairs in which a WEP is maximally true. The distance measure modulates the effect of distance from the prototype on the truth value of the WEP. This distance measure captures the intuition that WEPs vary in their strictness, e.g., intuitively, ‘impossible’ requires that the probability be very close to 0%, whereas ‘possible’ is felicitous in a much wider range of situations.

Thus, we define a prototype-based lexicon 𝔏_PT_. This lexicon associates each message *m* with a prototype *p*_*m*_ and a distance measure *d*_*m*_, so that the truth value of *m* in state *t* is:

$${𝔏}_{PT}\left(m,t\right)=\exp\left(-{\left(\frac{t-p_{m}}{d_{m}}\right)}^{2}\right)$$

Similarly to the threshold-based lexicon, prototypes and distance measures are treated as free parameters in the model, to be inferred from the data.

### Speaker Models

Given a lexicon, we may define two types of speakers in order to connect the hypothesised semantics to the data from the production experiment: a *literal* speaker and a *pragmatic* speaker. The literal speaker solely aims at being truthful, i.e., she prefers to produce true messages over false ones (𝔏_TH_), or messages with a higher truth value over messages with a lower truth value (𝔏_PT_).

We further assume that the available messages vary in their *salience*. Some WEPs come to mind more easily than others, as evidenced by their fluctuating production frequencies. To model effects of differential salience, we pair each message *m* with a salience value *P*_Sal_(*m*), which is treated as a free variable.

Thus, we may define a literal speaker *S*_lit_ as follows:

$$P_{S_{\text{lit}}}(m|t,𝔏)\propto P_{\text{Sal}}\left(m\right)𝔏\left(m,t\right)$$

This definition states that the probability that the literal speaker produces a message *m* in a state of affairs *t* is proportional to (i) the salience of *m*, and (ii) the truth value of *m* in *t*.

The literal speaker only cares about truthfulness. However, one of the central tenets of modern pragmatics is that speakers tend to behave *rationally*, i.e., they try to optimise the probability that their audience arrives at the correct interpretation (Grice, 1975). Given people’s cognitive limitations, we may plausibly expect that this form of rationality is bounded, so that speakers prefer to select the optimal message but occasionally deviate from this optimum.

Accordingly, we may define a pragmatic speaker *S*_prag_. The pragmatic speaker is truthful but also seeks to optimise the chance of coordination with a literal listener *L*_lit_. The literal listener, in turn, naively infers a state of affairs with a probability that is proportional to the truth value of the message in that state:

$$\begin{array}{l} P_{S_{\text{prag}}}(m|t,𝔏)\propto P_{\text{Sal}}\left(m\right)P_{L_{\text{lit}}}{\left(t|m,𝔏\right)}^{\lambda },\;\text{where}\\ P_{L_{\text{lit}}}(t|m,𝔏)\propto 𝔏\left(t,m\right) \end{array}$$

The parameter *λ* modulates the probability with which the speaker chooses the pragmatically optimal message, i.e., the message that is the most likely to receive the intended interpretation on the part of the listener (cf. Zaslavsky et al., 2020). We later investigate whether the inferred value for this parameter varies between participants with a high and low AQ.

The literal and pragmatic speaker models specify the probability of a message given a state. Our linking hypothesis is that this probability reflects the probability that a speaker would produce that message in that state, i.e., our hypothesis is that it approximates the corresponding production probability in Exp. 1.

The current speaker models assume perfect knowledge of the actual state of affairs. Though there may be circumstances in which this assumption is plausible, the use of WEPs is generally associated with uncertainty about the actual probability (e.g., Teigen & Brun, 2003). To model such uncertainty, we enrich the speaker models with a module representing the approximate perception of numerosity. While there may be other factors that cause uncertainty, the inaccurate perception of the number of red marbles is presumably the most prominent one in the context of our experiment.

### Number Perception

Speakers in the production experiment had to estimate the actual probability of drawing a red marble, i.e., the number of red marbles. The cognitive system used to estimate large numerosities is called the *Approximate Number System* (ANS) (Dehaene, 1997; Feigenson et al., 2004). It is well known that the estimates of the ANS are prone to error. In particular, the accuracy of the ANS decreases as the number to be estimated increases.

To model the accuracy of participants’ estimates, we define the confusion probability *P*_Cf_(*t*′|*t*) of perceiving the actual state of affairs *t* as *t*′. Since the visual displays in the production experiment were upper-bounded, *P*_Cf_(*t*′|*t*) is defined as the product of the probability *P*_ANS_(*t*′|*t*) of maintaining an approximate representation of the number *t* as *t*′ and the inverse probability *P*_ANS_(100 − *t*|100 − *t*′). These probabilities, in turn, are specified as follows:

$$\begin{array}{l} P_{\text{Cf}}\left(t^{\prime }|t\right)\propto P_{\text{ANS}}\left(t^{\prime }|t\right)P_{\text{ANS}}\left(100-t^{\prime }|100-t\right)\\ P_{\text{ANS}}\left(t^{\prime }|t\right)=\int_{t\prime -0.5}^{t\prime +0.5}\text{Gaussian}\left(x,\mu =t,\sigma =w\;t\right)dx \end{array}$$

The parameter *w* stands for *Weber’s fraction*, which represents the accuracy of participants’ estimates. To parametrise *w*, we carried out an experiment (Exp. 2) in which we presented 50 participants with the same types of displays used in the production experiment (Figure 2).^3^ Participants had to estimate the percentage of red marbles using a continuous slider. Each participant saw 25 vases with random proportions of red marbles. Based on the results of this experiment, we determined that the maximum likelihood estimate of the Weber fraction was *ŵ* = 0.35. We use this value in all production models.

We added the numerosity estimation module to our speaker models. If *P*_S_(*m*|*t*, 𝔏) is a speaker production rule, either literal or pragmatic, the production probabilities under approximate perception of the actual state are:

$$P_{S}^{\text{Cf}}\left(m|t,𝔏\right)\propto \sum_{t\prime \in T}P_{\text{Cf}}\left(t^{\prime }|t\right)P_{S}\left(m|t^{\prime },𝔏\right)$$

## MODEL COMPARISON

Taken together, we may distinguish four speaker models, varying the lexicon between threshold-based and prototype-based, and the speaker type between literal and pragmatic.^4^ All four models were implemented in Stan (Stan Development Team, 2018) to obtain samples from the posterior distribution over free parameter values conditioned on the data from the production experiment. For the purpose of model comparison, we split our dataset into a training set (the first 195 participants) and a test set (the remaining 60 participants). The four models were trained on the larger training set and were evaluated based on how well they explained the training set.

**Figure 4.**
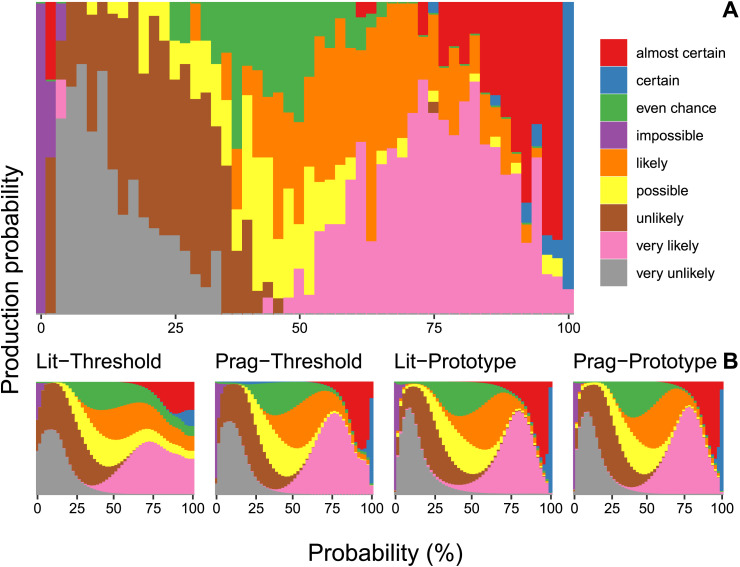
**(A) Production probabilities of a representative selection of the WEPs in our sample (Exp. 1). (B) Predicted production probabilities for each of the four speaker models.** Note that, for this figure, production probabilities are binned into bins consisting of two adjacent probabilities, except for probabilities 0 and 100.

To illustrate model performance, Figure 4 shows the data and posterior predictive distribution for a representative selection of the WEPs in our sample. (We visualised a subset of the WEPs to ensure that the figure remains readable.) This figure suggests that the literal threshold-based model offers a relatively poor approximation of the production data, while the other three models fare much better.

For proper statistical model comparison, we look at how well each model is able to predict the test dataset by calculating the expected log pointwise predictive density using the ‘elpd()’ function from the R package ‘loo’ (Vehtari et al., 2022). For this analysis, we included all WEPs in our sample (cf. Figure 3), and used the unbinned data and predictions. The model fits were statistically compared using the ‘loo_compare()’ function from the same package. Table 1 shows the outcome of this comparison.

**Table 1.** Differences in expected log pointwise predictive density relative to the optimal pragmatic prototype-based model (elpd_diff) and corresponding standard error of the difference (se_diff). The expected log pointwise predictive density is a measure of overall model fit, so the difference indicates how much worse the model predictions are compared to the pragmatic prototype-based model.

	elpd_diff	se_diff
prag-prototype	0.0	0.0
prag-threshold	−6.4	8.0
lit-prototype	−13.8	5.2
lit-threshold	−63.2	15.3

The table indicates that the pragmatic prototype-based model was the optimal one, but was not significantly better than the pragmatic threshold-based model, since the difference is smaller than corresponding standard error. By contrast, the pragmatic prototype-based model was superior to the other two models, since the difference in both cases was greater than twice the standard error. The comparable fit of the two pragmatic models shows that patterns of gradience and focality in the use of WEPs can be explained equally well within a threshold-based approach as within a prototype-based approach that directly encodes these patterns into the semantics of WEPs.

Before discussing these results, we turn to investigate the effects of AQ on the lambda parameter that modulates the probability with which the speaker chooses the pragmatically optimal message.

## AUTISM SPECTRUM QUOTIENT

After the production experiment, we asked all participants to fill out the Autism Spectrum Quotient test, which is a 50-question multiple choice questionnaire in which participants have to indicate if they agree or disagree with certain statements that pertain to traits that are often associated with ASD.

To investigate the effect of AQ on speaker behaviour, we divided participants based on whether their AQ was above or below the median AQ across all participants in our sample (i.e., 22), with participants at the median assigned to the high-AQ group. The average AQ of the high-AQ group was 26 (range: 22–36); of the low-AQ group 14 (range: 3–21). Due to computational limitations, we could not incorporate AQ as a continuous measure; hence, this analysis is inevitably coarse-grained.

To put these AQ values in perspective, Baron-Cohen et al. (2001) suggest that an AQ of 32 or higher is a reliable indicator of the presence of Autism Spectrum Disorder (ASD), since approximately 80% of their autistic participants had an AQ of at least 32, compared to only 2% of their neurotypical participants. Our high-AQ participants were mostly below this threshold, i.e., even though they exhibited autistic traits, they were generally not at risk of having ASD.

For this analysis, we focus on the pragmatic threshold-based model. Using STAN, we obtained samples from the posterior distribution over free parameter values conditioned on the data from the production experiment. We fit the model using the combined training and test datasets. Crucially, we fit different lambda parameters for the datasets from high-AQ and low-AQ participants.

By fitting the model on the entire dataset, rather than on the datasets from high-AQ and low-AQ participants separately, we ensure that all parameters except for the lambda parameter remain constant across both groups of participants, so that differences in the lambda parameter cannot be interpreted as statistical “spandrels” compensating for differences in other parameters. Reassuringly, the same pattern of results emerges if the model is in fact fit on both datasets separately.

**Figure 5.**
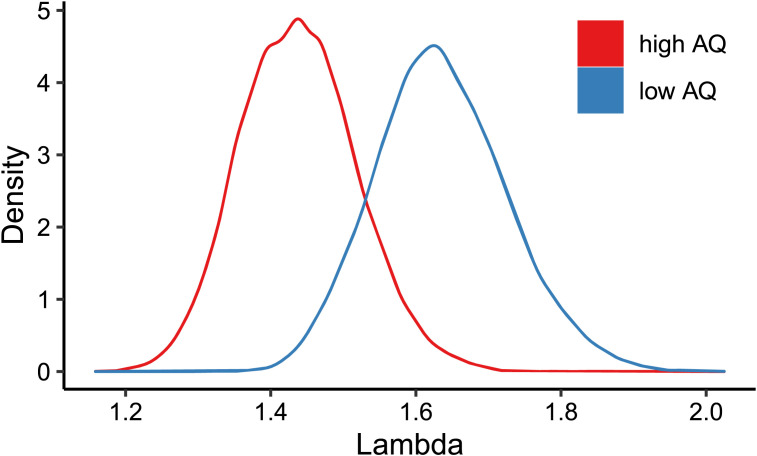
Density plot of the posterior estimates of the lambda parameter for high-AQ and low-AQ participants.

Figure 5 shows the posterior estimates of the lambda parameter for high-AQ and low-AQ participants. The mean estimated lambda parameter for high-AQ participants was 1.44; for low-AQ participants 1.64. A *t*-test indicated that this difference was significant (*t*(31520) = 206, *p* < .001). This analysis suggests that participants with a high AQ were less likely to select the pragmatically optimal message than participants with a low AQ.

## GENERAL DISCUSSION

People associate WEPs with gradient and focalised ranges on the probability scale. It has sometimes been concluded that these patterns of use must be reflected in the underlying semantics of WEPs (e.g., Bocklisch et al., 2012; Jaffe-Katz et al., 1989). Here, we have shown that this conclusion is unwarranted: data from a novel production experiment could be explained equally well on the basis of a truth-conditional approach that associates WEPs with crisp thresholds on the probability scale as on the basis of a prototype-based approach that directly encodes gradience and focality into the semantics of WEPs. Importantly, this equivalence only holds if the threshold-based approach is embedded in a probabilistic model that encodes perceptual limitations and goal-directed speech.

On a more abstract level, our results lend support to a modular approach that distinguishes between the conventional meaning of an expression and what a speaker who uses that expression conveys. This bifurcation between meaning and use has a number of important theoretical advantages (e.g., explaining entailment patterns, cf. Barwise & Cooper, 1981). In this paper, we provide further support by showing precisely how the lean meanings postulated by the threshold-based approach may lead to rich and complex patterns in language use.

One of the central insights of modern pragmatics is that speaker behaviour can be viewed, to a large extent, as rational, i.e., goal-oriented action. Recent probabilistic models provide a means to precisely quantify to what extent speakers behave rationally. Interestingly, Autism Spectrum Disorder (ASD) is said to be characterised, in part, by a pragmatic deficit. Hence, we intuited that this deficit might be reflected in the model parameters, specifically in a parameter that modulates the degree of rationality. We indeed find that participants with more autistic traits—as measured using the Autism Spectrum Quotient test—were estimated to have a significantly lower rationality parameter than participants with fewer autistic traits.

This observation provides an interesting counterpoint to earlier findings showing that participants with and without ASD are equally likely to derive *scalar inferences*, such as the inference from ‘some’ to ‘not all’ (e.g., Chevallier et al., 2010; Pijnacker et al., 2009; Su & Su, 2015). The derivation of scalar inferences is also assumed to be reliant on (the hearer’s assumption of) rational speaker behaviour (e.g., Geurts, 2010; Horn, 1972). How can this discrepancy be explained, i.e., why do people with and without ASD derive scalar inferences at equivalent rates but do people with more autistic traits behave less rationally in our production experiment? One possible explanation is that the pragmatic effects of autistic traits are too subtle to be brought out using coarse-grained tasks such as asking whether sentences like ‘Some dogs are mammals’ are true or false, but that these effects surface in more naturalistic contexts as exemplified by our production experiment (cf. van Tiel & Kissine, 2018).

At the same time, it should be noted that the connection between AQ and ASD is not uncontentious. First, a number of authors have argued that the AQ test is not an adequate predictor of the presence or absence of ASD (e.g., Ashwood et al., 2016; Lundqvist & Lindner, 2017). In particular, these studies show that the AQ thresholds used for identifying people at risk of having ASD substantially underestimate the actual prevalence of ASD. More problematically, it has recently been argued that there are important theoretical and practical problems associated with the construal of autism as a spectrum, i.e., as a collection of traits that, to a lesser degree, are also shared by the non-autistic population (Mottron & Bzdok, 2020; Sasson & Bottema-Beutel, 2022). Given these concerns, the current findings call for confirmation using gold-standard instruments such as the Autism Diagnostic Observation Schedule (Lord et al., 2000).

Our production experiment elicited various types of WEPs, including adjectival (e.g., ‘likely’) and nominal (e.g., ‘a good chance’) ones. The pragmatic model assumes that these WEPs compete with each other to the same degree. At the same time, we observed that about 40% of the participants consistently produced either adjectival or nominal WEPs. Consequently, adjectival WEPs were more likely to co-occur with other adjectival WEPs than with nominal ones, and vice versa, suggesting that expressions from the same part of speech compete with each other more strongly than with expressions from different parts of speech. An interesting direction for future research is to encode such differential levels of “alternativeness” into the model.

The communication of probability is of great importance in high-risk areas such as healthcare (e.g., Lipkus, 2007). Here, we have successfully implemented a computational model that explains the use of probability expressions while being firmly rooted in sound linguistic theory. We hope to have thereby contributed to a better understanding of the use and misuse of these expressions.

## ACKNOWLEDGMENTS

We thank the audience at the 9th Experimental Pragmatics Conference in Pavia for helpful feedback. We also thank our anonymous reviewers for raising important issues about WEPs in different parts of speech, and about the semantics/pragmatics divide in prototype theory. This research received financial support from German Research Council Grants FR 3482/2-1, KR 951/14-1, and SA 925/17-1, in part within Grant SPP 1727 (Xprag.de).

## AUTHOR CONTRIBUTIONS

All authors formulated the project, and designed and conducted the experiments. MF developed the probabilistic model. BvT and MF implemented and tested the model. All authors wrote the paper.

## Notes

^1^ A note on terminology: we use the term ‘meaning’ to narrowly refer to the conventional content of an expression rather than what someone who uses that expression conveys, i.e., to refer to *semantic* rather than *pragmatic* meaning.^2^ The Supporting Information contain an appendix with more details about the production experiment, as well as anonymised data and analysis files.^3^ The Supporting Information contain an appendix with more details about the numerosity estimation experiment, as well as anonymised data and analysis files.^4^ Note, in passing, that if we construe prototype-based meanings as reflections of patterns in language use, it seems redundant to assume that they enter into a further pragmatic reasoning process. Nevertheless, for completeness, we also consider the possibility of a pragmatic speaker using prototype-based meanings.

## Supplemental Materials

https://osf.io/khf3n
